# Concepts of good mental health and wellbeing in people with intellectual disability: Study protocol for a systematic review

**DOI:** 10.3389/fpsyt.2023.1148702

**Published:** 2023-04-06

**Authors:** Sophie Komenda, Nadine Brunevskaya, Paula Moritz, Sarah Jasmin Landskron, Irina Zrnic Novakovic, Sandra Oberleiter, Jana Wurzer, Brigitte Lueger-Schuster, Luis Salvador-Carulla, Elisabeth L. Zeilinger

**Affiliations:** ^1^Department of Clinical and Health Psychology, Faculty of Psychology, University of Vienna, Vienna, Austria; ^2^Division of Palliative Medicine, Department of Medicine I, Medical University of Vienna, Vienna, Austria; ^3^Department of Developmental and Educational Psychology, Faculty of Psychology, University of Vienna, Vienna, Austria; ^4^Division of Hematology and Hemostaseology, Department of Medicine I, Medical University of Vienna, Vienna, Austria; ^5^Health Research Institute, Faculty of Health, University of, Canberra, ACT, Australia

**Keywords:** assessment, definition, health equity, intellectual & developmental disabilities, intellectual disabilities, measurement, mental health, wellbeing

## Abstract

**Introduction:**

Good mental health is an indispensable aspect of good general health and different definitions of good mental health have been developed for the general population. However, it is not clear how these definitions can be applied to people with intellectual disabilities (ID), as they may have difficulties in many facets encompassed in existing definitions. Yet, people with ID can be mentally healthy or mentally ill just as people without ID.

**Objective:**

The aim of this systematic review is to summarize existing concepts, definitions, and measurement approaches of good mental health and wellbeing for people with ID.

**Methods:**

A comprehensive, systematic literature review will be conducted in 11 databases, including ASSIA, CINAHL, Cochrane Library, ERIC, MedRxiv, OSF preprints, ProQuest Dissertations & Theses Global, PsycINFO, PubMed, Scopus, and Web of Science. By including preprints and theses servers in the search strategy, we will also consider grey and unpublished literature. The quality of included studies will be rated using standardized checklists. All steps of the literature search, data extraction, and quality rating will be performed independently by two trained researchers. Disagreements will be resolved through discussion between these researchers and, if required, by consulting a third team member. In a narrative synthesis, existing approaches to good mental health and wellbeing for people with ID will be systematically described and linked to current research in mental health for people with and without ID.

**Discussion:**

The findings of this study will provide researchers and practitioners with an evidence-based overview of current approaches to good mental health and wellbeing of people with ID. We will explore and discuss the individual facets of the definitions, concepts, and measurement approaches and identify possible gaps which need to be addressed. This will strengthen future research on this topic and focus research activities on important and unresolved themes that need to be targeted to promote health equity for people with ID.

## 1. Introduction

Intellectual disability (ID) is characterized by limitations in intellectual functioning (IQ < 70) and in adaptive behavior originating during the developmental period (1). It is also known as Disorders of Intellectual Development in the 11th revision of the International Classification of Diseases (ICD-11) (2, 3), and as Intellectual Developmental Disorder in the fifth edition of the Diagnostic and Statistical Manual of Mental Disorders (DSM-5) (4). For the current study the terminology intellectual disability (ID) as defined by the American Association on Intellectual and Developmental Disabilities (AAIDD) (1) will be applied.

One of the most prominent definitions of good mental health is the one by the World Health Organization (WHO): “*a state of well-being in which every individual realizes his or her own potential, can cope with the normal stresses of life, can work productively and fruitfully, and is able to make a contribution to her or his community*” (5). Another common definition of good mental health, especially in the opinion of laypersons, is the mere absence of mental disorder or mental illness. However, presently the majority of scholars agree that this approach is not adequate to capture the complexity of good mental health (e.g., Ref. (6)). It is argued that mental illness and mental health represent two correlated but distinct aspects (6). Traditionally, two approaches to good mental health are discussed: the hedonic and the eudaimonic approach. The hedonic approach includes primarily positive affect, whereas the eudaimonic approach includes optimal functioning in everyday life and leading a fulfilling and purposeful life (6–8). Current definitions of good mental health for the general population include both aspects.

Good mental health contributes to a better quality of life and enables social participation and inclusion for people with and without ID. Research has shown that psychosocial functioning can be better predicted by assessing both mental health and mental illness rather than by assessing only one of these two aspects alone (e.g., Ref. (6)). This supports the importance of conceptualizing and assessing mental health with the same emphasis and relevance as mental illness. The term mental health has a positive connotation in itself, but is often used as a euphemism for mental illness. To emphasize the inherently positive meaning of mental health and to distinguish it from the term mental illness, the term good mental health will be used in the present work.

Generally, it is not sufficiently clear how definitions such as the WHO’s can be meaningfully applied when it comes to people with ID, as people with ID may have difficulties in almost all of the mentioned concepts: They may not be able to realize their potential, mostly have limited coping strategies, and often limited skills and opportunities to work productively and to contribute to their community. Further definitions of good mental health, such as that proposed by the *Committee on Ethical Issues by the European Psychiatric Association* (9), by Vaillant (10), or by Manwell and colleagues (11), share the same limitations with regard to people with ID. However, this may also be a function of the model of disability in use. Considering the human rights model or the social model of disability (12), existing mental health definitions for the general population could also be applicable to people with ID. This, however, raises the following question: How is good mental health conceptualized for people with ID, and which definitions are currently applied in the scientific literature?

More than 20 years ago, a paper published following the inaugural meeting of the *Mental Health Special Interest Research Group* (MH/SIRG) of the *International Association for the Scientific Study of Intellectual Disability* (IASSID) described extremely diverse perspectives on theoretical frameworks concerning mental health problems, assessment, and treatment in people with ID (13). It was recognized that there were no substantial frameworks to conceptualize good mental health in people with ID. Since then, there have been numerous efforts focusing on good mental health, wellbeing, and other positive psychology constructs in people with ID (e.g., Refs. (14–16)). A recent systematic review summarized research on positive psychology constructs for people with ID, including aspects such as job satisfaction, self-determination, or character strengths (17). Also, assessment instruments related to good mental health and wellbeing have been developed, such as the *Personal Wellbeing Index-Intellectual Disability* (PWI-ID) (18). Established assessment tools can be valuable in the search for theories and conceptualizations of good mental health, as they are usually based on a theory or assumption about the construct to be assessed. They can provide particularly application-oriented approaches to good mental health and will thus be included in the review.

To summarize, a variety of research related to the conceptualization of good mental health and well-being for people with ID has been conducted in recent decades. However, a systematic review specifically targeting good mental health as an outcome has not yet been realized. The main objective of this systematic review is to summarize existing concepts, definitions, and measurement approaches of good mental health and wellbeing for people with ID.

## 2. Methods and analysis

This review will be conducted and reported according to the Preferred Reporting Items for Systematic Reviews and Meta-Analyses (PRISMA) statement (19). The present review protocol has been developed according to the PRISMA guidelines for protocols (PRISMA-P) (20, 21). The review was submitted for registration in the *International Prospective Register of Systematic Reviews* (PROSPERO) with registration number: CRD42023385396. Potential amendments made in the course of the review will be included in the PROSPERO protocol along with an accompanying rationale. Any changes to the original protocol will also be mentioned and explained in the final publication of the study results.

### 2.1. Search strategy

**Figure 1:**
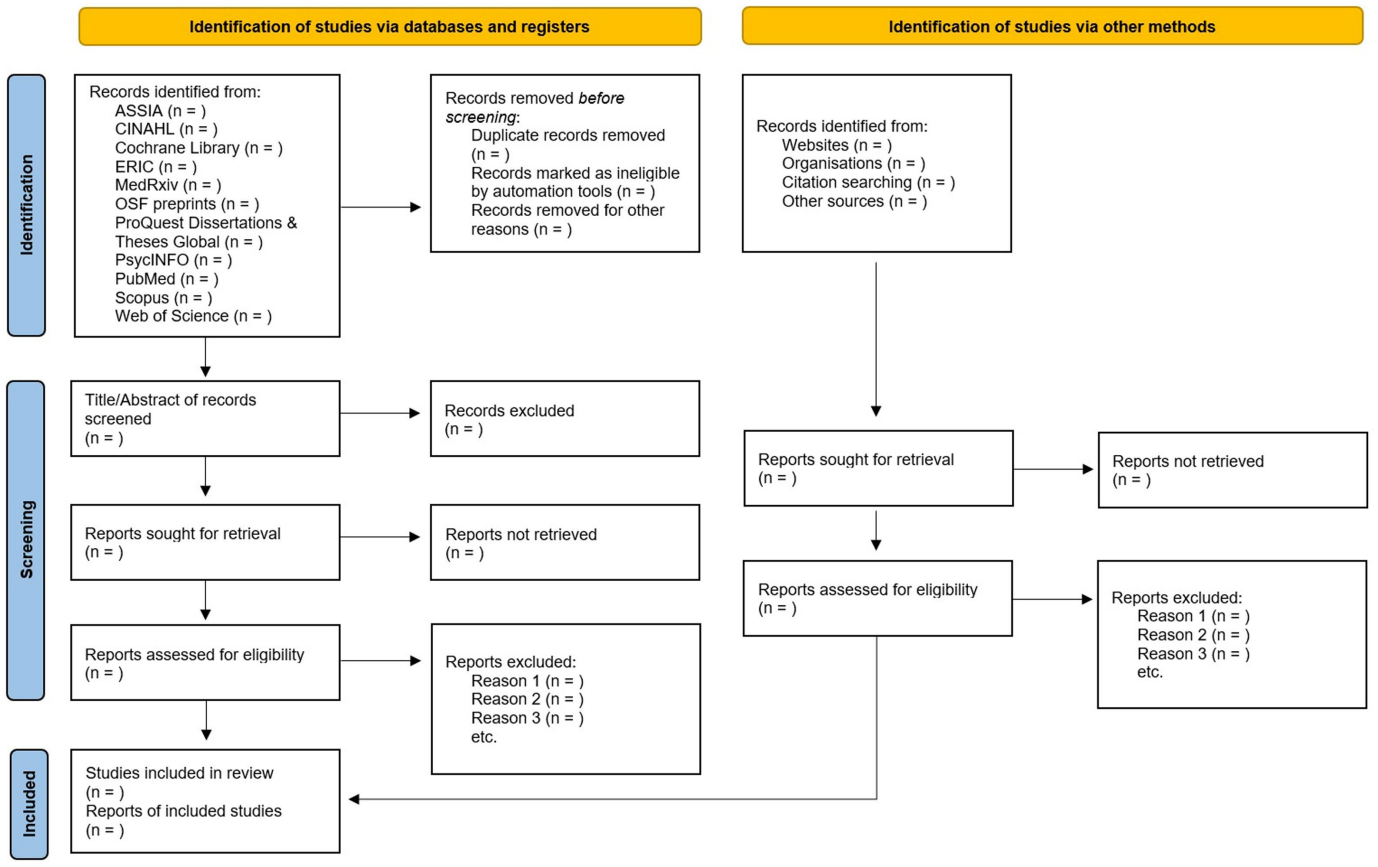
PRISMA flowchart.

A comprehensive search strategy will be applied and carried out between February 2023 and May 2023. Figure 1 shows the planned search strategy using the PRISMA flowchart. We will use 11 databases, including ASSIA, CINAHL, Cochrane Library, Education Collection, MedRxiv, OSF preprints, ProQuest Dissertations & Theses Global, PsycINFO, PubMed, Scopus, and Web of Science. The development of the search string is depicted in Table 1. We will include various synonyms or related concepts for the following terms (i) intellectual disability, (ii) mental health, (iii) definition, and (iv) assessment. Although mental health and wellbeing are not synonyms and may be regarded as different conceptual frameworks (7), this work will include wellbeing alongside mental health, as it features prominently in the current definitions most often referred to when discussing good mental health.

**Table 1:** Search strategy.

Terms of interest	(i) Intellectual disability	(ii) Mental health	(iii) Definition	(iv) Measurement
Related terms and synonyms	learning disability, intellectual developmental disorder, disorders of intellectual development, mental retardation, mental deficiency	wellbeing, well-being	defining, concept, conceptualization, model, modeling	assessment, assessing, measuring, measure, scale
Combined and truncated	“intellectual^*^ disab^*^” OR “learning disabilit^*^” OR (development^*^ AND disorder^*^ AND intellectual) OR (mental AND (retard^*^ OR deficien^*^))	(mental AND health) OR wellbeing OR well-being	defin^*^ OR concept^*^ OR model*	measure^*^ OR assess^*^ OR scale^*^
Search string for PubMed	(“intellectual^*^ disab^*^”[Title] OR “learning disabilit^*^”[Title] OR (development^*^[Title] AND disorder^*^[Title] AND intellectual[Title]) OR (mental[Title] AND (retard^*^[Title] OR deficien^*^[Title]))) AND ((mental[Title] AND health[Title]) OR (wellbeing[Title] OR well-being[Title])) AND ((defin^*^[Title/Abstract] OR concept^*^[Title/Abstract] OR model^*^[Title/Abstract]) OR (measure^*^[Title/Abstract] OR assess^*^[Title/Abstract] OR scale^*^[Title/Abstract]))			

To design the search string, the four terms mentioned will be combined using Boolean as follows: 1 AND 2 AND (3 OR 4). The first two terms are our main focus and we expect both terms, respectively their synonyms, to be mentioned in the title of the relevant records. Terms three and four delve more into detail of our research question and will therefore be searched in the title and abstract of the records. The search string for PubMed has been built and is available in Table 1. This search string will be adapted to other electronic databases used in this study. To account for grey and unpublished literature, we will include the preprint servers MedRxiv and OSF preprints as well as ProQuest Dissertations & Theses Global in our search strategy. Furthermore, we will follow up on meeting abstracts. References of papers meeting the inclusion criteria will be hand-searched.

### 2.2. Inclusion/exclusion criteria

The following criteria will be used in the review process: Inclusion criteria: (i) studies need to focus on good mental health or wellbeing in adults with ID, (ii) studies need to focus on either a definition, conceptualization or model of good mental health/wellbeing or provide a measurement approach of good mental health/wellbeing. The rationale for including the term wellbeing alongside mental health in the search strategy relates to its prominent use in current definitions, e.g., the one by the WHO (5). However, to keep the focus specifically on good mental health in adults with ID, no other related constructs are included in our search. Therefore, the following exclusion criteria are applied: (i) studies focusing on related concepts, but not targeting good mental health or wellbeing will be excluded, e.g., studies focusing on quality of life or happiness without conceptual relation to good mental health,(ii) studies focusing solely on children or adolescents with ID will also be excluded.

We will include all studies, irrespective of their design. No limitations related to language or year of publication will be set. We will update the search prior to the final synthesis to include the most recent studies.

### 2.3. Study selection

Duplicates will be removed by one reviewer. All remaining records will be reviewed by two team members independently, i.e., blinded to each other’s decisions. Firstly, only titles and abstracts will be screened. Secondly, the full texts of included records will be assessed and evaluated according to our inclusion/exclusion criteria. In both stages, disagreements between reviewers will be discussed until agreement is reached. In the case of non-agreement, a third team member will be included in the discussion. This will be either SK or ELZ, who both have a background in mental health and ID as well as experience in conducting systematic reviews (e.g., Refs. (22–26)). Decisions will be recorded in Excel spreadsheets.

### 2.4. Data extraction

We will extract two types of information: First, at the study level and second, at the outcome level. Information on study level will comprise descriptive information about included studies, such as publication year, study location, study aim, sample description (if any sample was used, including sample size, gender distribution, mean age, and level of ID), study design, and quality rating of the study. Information on outcome level will include descriptive information on the concepts, definitions, or measurements related to good mental health, that are reported or evaluated in the included studies. Our main outcome will be the description of the concept/measurements, i.e., which aspects are included in the conceptualization of good mental health or wellbeing for people with ID. Further information extracted will comprise the following facets: is the outcome of interest a definition/concept/measurement approach, which population is targeted (age, gender, severity of ID), does the outcome relate to an established concept/definition (e.g., WHO definition of good mental health), how are ID-specific aspects included, what is the intended use of the outcome, what are further findings of the study, and which study limitations are mentioned. This information will provide a comprehensive overview of each included study and a description of the conceptualization of good mental health or wellbeing in each case.

The extraction of all relevant data will be done *via* standardized and piloted Excel spreadsheets. Comparably to the study selection process, data extraction will be done by two team members independently, i.e., blinded to each other’s decisions. In the case of disagreement, dissonances will be discussed until agreement is reached. In the case of non-agreement, a third team member will be included in discussion. If we find that information relevant to our research goal is missing from a study, we will contact the study authors to obtain this information. For this, we will send an initial request and a reminder after 2 weeks. If we do not receive a response 2 weeks after the reminder and the study is not usable without the requested information, we will exclude the study.

### 2.5. Risk of bias and quality assessment

To provide a quality assessment of the included studies, we will use the Mixed Methods Analysis Tool, version 2018 (MMAT; (27)). This tool is particularly suitable for the present study as it can be applied to different types of study designs, including quantitative, qualitative and mixed-methods studies. For opinion pieces, that are not covered by the MMAT, we will apply the *Checklist for Text and Opinion*, a critical appraisal tool by the Joanna Briggs Institute (28). We assume, that publication bias will not be of central importance for our study, since we aim to summarize existing theoretical concepts, definitions and measurement approaches and do not focus on specific outcomes that may be over−/underreported in published research.

All quality ratings will be done by two reviewers independently. In the case of disagreement, dissonances will be discussed until agreement is reached. In the case of non-agreement, a third team member will be included in the discussion. Initial interrater agreement will be determined by percentage agreement. The quality ratings of the studies will go into the final appraisal of the quality of available research.

### 2.6. Strategy for data synthesis

A narrative synthesis will be conducted, following the procedures established in the *Guidance on the Conduct of Narrative Synthesis in Systematic Reviews* (29). The four steps of conducting a narrative synthesis comprise: (i) developing a theoretical model, (ii) developing a preliminary synthesis, (iii) exploring relationships in the data, and (iv) assessing the robustness of the synthesis product. The last step will also encompass an assessment of the strengths of the body of evidence.

Study characteristics will be presented in a table along with their quality ratings. For an overview, key aspects of outcomes will also be presented in tables. The main results related to the outcome of interest, i.e., definitions/concepts/measurements of good mental health, will be narratively summarized and related to current aspects of mental health research for people with and without ID. We will also provide numeric results on the frequency of concepts or measurement approaches used in included studies. If a sufficient number of primary studies can be included, we will also synthesize our results by subgroups in terms of age of the target group, severity of ID, and type of ID.

Potential pitfalls in this work may include insufficient literature on good mental health for people with ID. It is not certain that we will find enough published work on the conceptualization of good mental health. However, our pilot searches concluded that there is sufficient research related to either good mental health or wellbeing to constitute a meaningful review and also to discuss potential gaps in the literature, which may very well relate to a lack of conceptualization of good mental health for people with ID. Patient and Public Involvement.

This review was designed without involvement of people with ID or other members of the public. However, the results of this study will be used as a starting point for a participatory research project. We will conduct focus groups and interviews with people with ID and other experts on mental health for people with ID. People with ID will be a significant part of the projects advisory board.

## 3. Discussion

Good mental health is an indispensable aspect of good general health. People with ID can be mentally healthy and mentally ill just like people without ID. However, current definitions of good mental health cannot be adequately applied to people with ID. Based on definitions developed for the general population, it is impossible for people with ID to be considered mentally healthy, highlighting the need for concepts specifically tailored to people with ID. Moreover, mental health promotion and mental health care services for people with ID have to be based on theoretical models and definitions of good mental health and models how to maintain or regain it in this specific population (30, 31). Without an appropriate concept of good mental health, this population is excluded from high-quality mental health promotion strategies and preventive measures.

In recent decades, there has been a growing amount of research targeting good mental health, wellbeing, and aspects of positive psychology, both for the general population as well as for people with ID. While a synthesis of the literature on concepts of good mental health has been conducted for the general population (32), such a synthesis of concepts, definitions and measurement approaches to capture good mental health in people with ID is lacking. The present review will therefore comprehensively summarize the existing approaches to good mental health and wellbeing for people with ID. We will provide researchers and practitioners with an evidence-based overview of the available approaches, their inherent facets, and potential gaps that need to be addressed, which will strengthen future research on this topic and focus research activities on important issues that need to be targeted in order to promote health equity for people with ID.

## Author contributions

EZ and SK conceived the study and drafted the manuscript. NB, SL, IZN, and SO contributed to the development of the selection criteria, the risk of bias assessment strategy, and data extraction criteria. PM provided expertise on intellectual disability. BL**-**S and LS**-**C provided mental health expertise. EZ, SK, and JW designed and tested the search strategy. The guarantor of the review is EZ. All authors contributed to the article and approved the submitted version.

## Funding

This research was funded by the Austrian Science Fund (FWF), grant no. ESP 116. For the purpose of open access, the author has applied a CC BY public copyright license to any Author Accepted Manuscript version arising from this submission.

## Conflict of interest

The authors declare that the research was conducted in the absence of any commercial or financial relationships that could be construed as a potential conflict of interest.

## Publisher’s note

All claims expressed in this article are solely those of the authors and do not necessarily represent those of their affiliated organizations, or those of the publisher, the editors and the reviewers. Any product that may be evaluated in this article, or claim that may be made by its manufacturer, is not guaranteed or endorsed by the publisher.
